# Differences in antigenic sites and other functional regions between genotype A and G mumps virus surface proteins

**DOI:** 10.1038/s41598-018-31630-z

**Published:** 2018-09-06

**Authors:** Sigrid Gouma, Tessa Vermeire, Steven Van Gucht, Lennart Martens, Veronik Hutse, Jeroen Cremer, Paul A. Rota, Geert Leroux-Roels, Marion Koopmans, Rob van Binnendijk, Elien Vandermarliere

**Affiliations:** 1 Centre for Infectious Disease Control, RIVM, Bilthoven, The Netherlands; 2 Department of Viroscience, Erasmus MC, Rotterdam, The Netherlands; 3 Microbiology Department, Perelman School of Medicine, University of Pennsylvania, Philadelphia, USA; 4 National Reference Centre for Measles, Mumps and Rubella, Sciensano, Brussels, Belgium; 5 Department of Biochemistry, Ghent University, Ghent, Belgium; 6 VIB-UGent Center for Medical Biotechnology, VIB, Ghent, Belgium; 7 National Center for Immunization and Respiratory Diseases, Centers for Disease Control and Prevention (CDC), Atlanta, USA; 8 Center for Vaccinology, Ghent University, Ghent, Belgium

## Abstract

The surface proteins of the mumps virus, the fusion protein (F) and haemagglutinin-neuraminidase (HN), are key factors in mumps pathogenesis and are important targets for the immune response during mumps virus infection. We compared the predicted amino acid sequences of the F and HN genes from Dutch mumps virus samples from the pre-vaccine era (1957–1982) with mumps virus genotype G strains (from 2004 onwards). Genotype G is the most frequently detected mumps genotype in recent outbreaks in vaccinated communities, especially in Western Europe, the USA and Japan. Amino acid differences between the Jeryl Lynn vaccine strains (genotype A) and genotype G strains were predominantly located in known B-cell epitopes and in N-linked glycosylation sites on the HN protein. There were eight variable amino acid positions specific to genotype A or genotype G sequences in five known B-cell epitopes of the HN protein. These differences may account for the reported antigenic differences between Jeryl Lynn and genotype G strains. We also found amino acid differences in and near sites on the HN protein that have been reported to play a role in mumps virus pathogenesis. These differences may contribute to the occurrence of genotype G outbreaks in vaccinated communities.

## Introduction

Mumps is a very contagious childhood disease that is caused by the mumps virus, a member of the *Paramyxoviridae* family. The infection generally affects the parotid glands, which leads to the most characteristic symptom, a unilateral or bilateral swelling of these salivary glands. The mumps virus can also cause inflammation of the testis, ovaries, pancreas or meninges and lead to complications such as infertility or deafness^1,2^. Since the introduction of the measles, mumps and rubella (MMR) vaccine in many national immunization programs, mumps incidence has dramatically decreased. However, in the last decade, several mumps outbreaks among vaccinated young adults have been reported^3–7^.

These recent outbreaks may be due to waning of vaccine-induced immunity over time. Waning immunity is the most likely hypothesis, because most of the outbreaks affected adolescents who received their last MMR dose more than 10 years before the outbreaks, and because (neutralizing) serum antibodies were still detected prior to mumps virus exposure^8–13^. Another possible contributing factor for these outbreaks is strain variation^14–16^. Antigenic differences between the currently circulating wild-type virus and the vaccine virus may reduce recognition by vaccine-induced antibodies. Mumps viruses are divided into multiple genotypes which are defined based on the nucleotide sequence of the small hydrophobic (SH) gene^17^. The most frequently used vaccine is the Jeryl Lynn vaccine which consists of a mixture of two mumps virus strains that both belong to genotype A, whereas the most frequently detected mumps genotype in recent outbreaks around the world with genotype information is genotype G^7,18–22^. Although vaccine-induced antibodies neutralize genotype G strains, the level of neutralization is lower than for the vaccine strain (Rubin *et al*. 2008; Dayan & Rubin 2008; Gouma *et al*. 2016)^12,23,24^. This raises questions about the biological consequences of amino acid differences between genotype G and vaccine strains at sites that are important for immune recognition and pathogenesis. In addition, *in silico* analyses suggested differences in predicted B-cell and T-cell epitopes, but these studies did not focus on genotype G^25,26^. In this study, we compared the amino acid sequences from genotype A vaccine strains sequences and from genotype G. We focused on the fusion (F) and the haemagglutinin-neuraminidase (HN) proteins which are the main surface proteins of the mumps virus and are important for fusion, viral entry, and B-cell mediated antibody responses. All amino acid differences were also studied in the context of the protein structure. We found differences in known B-cell epitope regions and N-glycosylation sites, which could reduce immunological responses induced by the Jeryl Lynn vaccine. Additionally, differences were observed in regions that may play a role in mumps virus pathogenesis.

## Results

### Phylogenetic analysis

**Figure 1:**
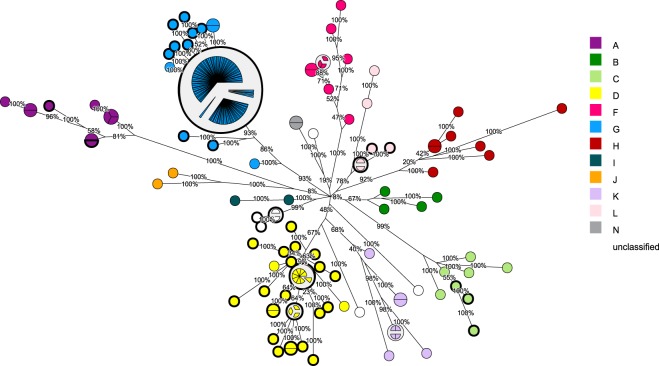
Maximum parsimony tree based on SH gene sequences. Representation of classification of 46 mumps virus strains from the pre-vaccination era and 110 recent mumps virus strains, all indicated by thick lines. Percentages indicate bootstrap values (1000 replicates). All but one mumps virus strain were collected from Dutch patients, the non-Dutch strain was isolated from a patient from Albany, USA in 1954. Genotypes are labeled by color. Mumps virus sequences retrieved from GenBank (n = 78), including WHO reference strains (n = 27), are included in the phylogenetic tree and are indicated by thin lines.

A phylogenetic analysis based on the SH gene sequences was performed to determine the genotypes of the samples from recent outbreaks in the Netherlands (n = 110), as previously described^27^, and from a collection of historic wild type strains isolated from Dutch patients since 1954 (n = 46) (Fig. 1). The strains from the recent outbreaks are classified as genotype G which is indeed the currently circulating genotype. Two historic wild type strains belong to genotype A, including a strain that is identical to MuVi/Boston.USA/0.45 “Enders”, the mumps prototype strain that was isolated from a patient in the USA. These two strains were isolated in 1954 and 1962, respectively. Three historic wild type strains belonging to genotype C were isolated from patients in 1980–1981. The majority of the historic wild type strains (n = 33) belong to genotype D and were isolated from patients (from 1961 to 1982), which indicates that this genotype circulated for several decades in the Netherlands before the introduction of the MMR vaccine. Four historic wild type strains belong to genotype L and were isolated from patients between 1957 and 1964. The four remaining historic wild type strains were isolated from patients between 1962 and 1964 and do not cluster with any of the genotypes as defined by the reference strains, which suggests a genotype that has not yet been described or may be extinct. Based on the SH gene sequences, these latter strains cluster together.

### Analysis of the F protein

F protein amino acid sequences from genotypes A and G were compared at sequence level and additionally at structure level, by mapping sequence variation on homology models. The homology model of the F protein was built using PDB-entry 4GIP, which is the F protein of the parainfluenza 5 virus (49% sequence identity). For the mapping of the sequence variation, Scop3D was used^28^, which is a tool designed to map sequence variation onto protein structure. Here, we used this tool to identify differences in residues on positions that are either specific to genotype A or genotype G strains. Because the F protein undergoes a large conformational change during the fusion process, it can adopt a pre- or post-fusion conformation^29^. In this work, we refer only to the pre-fusion conformation as this is the most important one for B-cell immunity.

**Figure 2:**
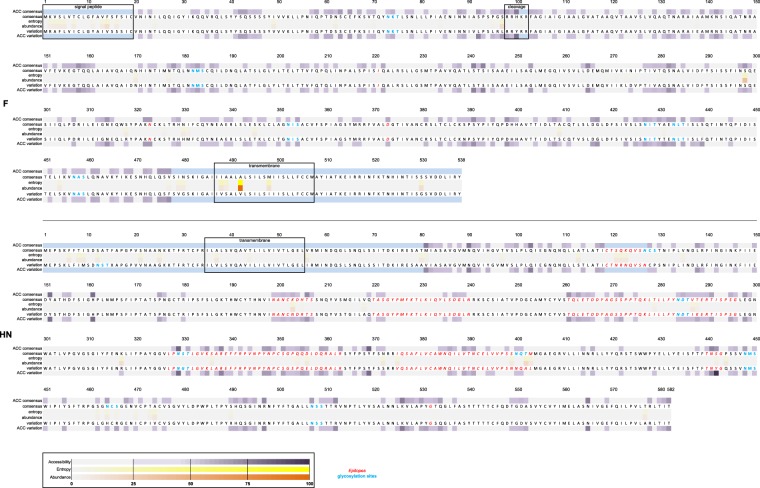
Overview of the consensus and the variable sequences of the F and HN proteins. Variation sequence shows all changes observed for both genotypes. Accessibility for the consensus (ACC consensus) and for the variation sequence (ACC variation), entropy and abundance are expressed as percentages and indicated by colors, indicated by the legend at the bottom. Epitope regions (italic, red) and glycosylation patterns (bold, blue) are indicated on the sequences. Parts of the structures that were missing in the protein models are indicated with the light blue regions. The upper part represents the F protein, the lower part the HN protein.

For the F protein, we found a total of 58 amino acid positions that show variation among the analyzed sequences of genotype A and G (Table 1). In a first analysis, only genotype A and G were compared. Only one position (position 2; Table 1, F row 1) is variable across both genotype A and genotype G sequences. Eight out of 58 positions are completely different between the genotype A and G strains, *i*.*e*. all genotype A sequences have a certain residue at that position, whereas all genotype G sequences present another residue (Table 1, F row 2). Of these genotype-specific positions, five are located in the signal peptide (Table 2, Fig. 2) while the other three positions are not located in a predicted or defined functional region.

**Table 1:** Variable positions found in the F and HN protein of genotype A and genotype G mumps virus sequences.

	Variable positions	F	HN
Comparison between A and G only	shared between genotype A and genotype G sequences	2	288*, 336*, 462*, 466*
	different between all genotype A and all genotype G sequences	3, 4*, 5*, 7*, 16*, 318*, 409, 454*	6, 9, 12, 21*, 56, 121, 122, 123, 287, 372, 375, 399, 444*, 577
	in subset of genotype A sequences only	70*, 95*, 477*, 489	44*, 81, 218, 279*, 464, 473*, 490*
	in subset of genotype G sequences only	62, 69, 84, 91, 92*, 138*, 195*, 269, 271, 498	13, 15, 25*, 130, 203, 353*, 402, 474, 533
Comparison with genotype A or G and historic genotypes	genotype A specific	13, 49, 345, 480*	8*, 80, 356
	genotype G specific	170, 330, 479, 488	113, 403
	genotype A specific, but only in a subset of the sequences	11, 24*, 177, 275, 326*, 331, 431	135, 205, 214*, 354*, 442*, 470, 552
	genotype G specific, but only in a subset of the sequences	14*, 96*, 97*, 100, 101, 115*, 141, 209*, 273, 274, 280, 298, 317*, 350, 389, 413, 425, 439*, 492, 530	37, 63, 94, 97*, 129*, 153, 317, 330
potentially located in B-cell epitopes		none	113, 121, 122, 123, 129, 130, 203, 205, 330, 336, 353, 354, 356, 375, 399, 402, 403, 442, 533

Underlined positions are surface-exposed residues in the variable situation.

*Non-conservative amino acid substitution.

**Table 2:** Functional regions of the F and HN protein.

Protein	Region	Residue position^a^
F	N-linked glycosylation pattern	73–75; 182–184; 352–354; 427–429; 433–435; 457–459
F	Known B-cell epitope	221, 323, 373
F	Fusion promotion	91; 195; 383
F	Cleavage site	98–102
F	Neurovirulence	91
HN	N-linked glycosylation pattern	12–14; 127–129; 176–178; 284–286; 329–331; 400–402; 448–450; 464–466; 507–509; 514–516
HN	Known B-cell epitope	113–130; 199–207; 220–240; 261–266; 327–363; 375–403; 440–443; 533
HN	Known T-cell epitope	279; 287
HN	Fusion promotion	82; 89; 96; 98; 102; 104; 111; 118; 222; 226; 228; 230; 567; 571
HN	Receptor binding	162; 175; 226; 228; 230; 335; 407; 422; 530; 531; 533; 540; 566; 567; 575
HN	Neuraminidase activity	180; 204; 239; 264; 268; 303; 407; 422; 466; 512; 540; 551; 561
HN	Ca^2+^-binding	268; 270; 272; 302
HN	Neurovirulence	360; 466

^a^F protein numbering is based on accession number JN012242; HN protein numbering is based on accession number ABY81903. Regions and residue positions are based on references^26,31,36,58–66^.

Four positions show a difference in a subset of the genotype A sequences when compared to genotype G (Table 1, F row 3). These four positions are not located in any specific functional region. Inversely, 10 positions were found to show a difference only in a subset of genotype G sequences, when compared to genotype A (Table 1, F row 4). Of these, two positions (positions 91 and 195) are linked to a fusion promotion site.

**Figure 3:**
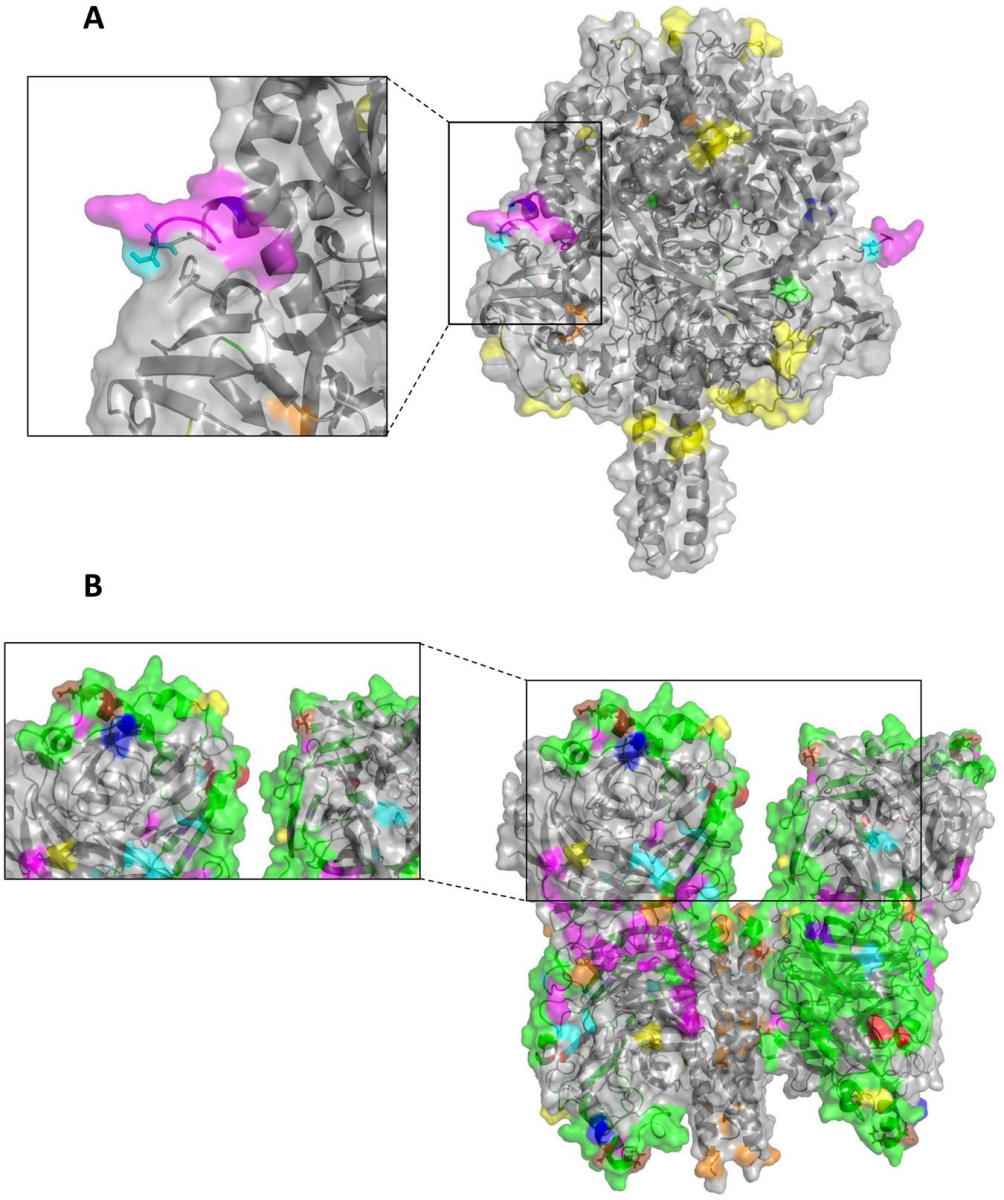
Overview of important variable positions on both the HN and F protein, as described in the literature. (**A**) The F protein important functional regions with glycosylation sites (yellow), fusion promotion sites (orange), cleavage site (pink), neurovirulence (dark blue) and known B-cell epitope regions (green) mapped on the pre-fusion structure. Zoom is on the variation at position 97 (cyan blue) near the cleavage site (pink). (**B**) The HN protein with glycosylation sites (yellow), fusion promotion sites (orange), receptor-binding regions (pink), neuraminidase activity regions (cyan blue), Ca^2+^-binding sites (red), neurovirulence regions (dark blue), known T-cell epitope (purple) and known B-cell epitope regions (green) mapped on the structure. Zoom is on the variations at positions 354, 356 and 442 (brown positions).

When the variable positions for each mumps virus strain were further compared with the historic wild type sequences also, four positions were found to be specific for both genotype A and genotype G (Table 1, F row 5 and 6). Seven positions for genotype A were found to vary only within genotype A sequences (Table 1; F row 7). No positions are related to specific functional regions. When the same comparison was made for the genotype G sequences, 20 positions were found to be specific for the genotype G sequences in our study (Table 1, F row 8). Four positions (positions 96, 97, 100 and 101) are related to the cleavage site (Table 1, Table 2, Figs 2, 3A). Of these four positions, the variation at position 97 (S → L) is observed in 19 out of 118 (16%) sequences (Figs 2, 3A). Furthermore, position 97 is surface exposed (56% solvent accessible; Fig. 2).

### Analysis of the HN protein

The same approach was used to analyze the HN protein amino acid sequences. The homology model of the mumps HN protein is based on the HN protein of the parainfluenza 5 virus (PDB entry 1Z4V; 46% sequence identity). During the course of our analyses, the structure of HN from mumps was solved via X-ray crystallography (PDB entry 5B2C^30^). Our homology model used in the analyses was similar to this experimentally solved structure (root mean square deviation (RMSD) of C_α_ = 1.20 Å).

We found 54 positions that show a difference in the analyzed HN sequences of genotypes A and G. Four positions (288, 336, 462, 466) are variable across both genotype A and genotype G sequences (Table 1, HN row 1), of which positions 462 and 466 cluster together and occur two times within the same sequence (distance C_α_ - C_α_ = 4.8 Å). Position 466 (S → R) is located in an N-glycosylation site (464–466) and the variation leads to the loss of this N-glycosylation site. The S → R variant results in a major change in the physicochemical properties of the side chain. Position 336 is located in a known B-cell epitope region. In 14 positions, the genotype A and genotype G sequences differ completely (Table 1, HN row 2). One of these 14 positions is located in an N-glycosylation site (position 12 in N-glycosylation site 12–14; Table 2, Fig. 2). Five other positions are situated in two different known B-cell epitopes^31^ (B-cell epitopes 113–130 and 375–403, Table 2, Fig. 2). One position (287) is linked to a known T-cell epitope (region 265–295)^25^. Six of these 14 positions could be mapped on the structure. None of the positions cluster together. The variation at position 287 is surface exposed (rSAS = 39%; Fig. 2).

Additionally, seven and nine positions show variation in a number of genotype A or genotype G sequences, respectively (Table 1, HN rows 3 and 4). Of the seven variable positions for genotype A, position 464 is situated in an N-glycosylation site (464–466; Table 2, Fig. 2). For the nine positions variable in some genotype G sequences, two positions are located in an N-glycosylation site (positions 13 and 402 in N-glycosylation sites 12–14 and 400–402, respectively; Table 2, Fig. 2). Four positions are located in five different known B-cell epitopes^26,31–33^ (Table 1, HN row Table 2, Fig. 2).

When the comparison of the variable positions was also made against the historic wild type strains, three and two positions that differ completely between genotype A and G, are specific for either genotype A or G (Table 1, HN rows 5 and 6). The variations at positions 113 and 356 are surface exposed (rSAS = 40% and 54% respectively; Fig. 2). Position 113 is not surface exposed in the consensus situation (rSAS = 23%, Fig. 2). Three (positions 205, 354 and 442) out of seven positions variable only in genotype A sequences, (Table 1; HN row 7) are located in known B-cell epitopes^26,31–33^ (epitope regions 199–207, 327–363 and 440–443). Eight positions are only variable in genotype G sequences when also compared to the historic wild type sequences, including positions 129 and 330, which are located in known B-cell epitopes^26,31–33^ (Table 1, HN row 8, Fig. 2). These positions are also located in an N-glycosylation site (N-glycosylation sites 127–129 and 329–331). Only position 129 is surface exposed in the variable situation (rSAS = 30% versus 16%).

Taken together, of the 20 positions that are specific for either of the two genotypes, eight positions (positions 113, 129, 205, 330, 354, 356, 403 and 442) are located in five different known B-cell epitopes^26,31–33^ (B-cell epitopes 113–130, 199–207, 327–363, 375–403 and 440–443; Table 1, Table 2, Fig. 2)

Several variable positions clustered together when analyzed on the protein structure. Positions 336 and 399 cluster together (distance C_α_ - C_α_ = 2.7 Å) and occur together in six sequences. Positions 354 and 356 also cluster together (distance C_α_ - C_α_ = 3.3 Å) and coexist in five sequences (Fig. 3B). Positions 464, 473 and 474 are located close to each other (distance C_α_ - C_α_ of 464 and 473 = 9.5 Å; distance C_α_ - C_α_ of 464 and 474 = 8.4 Å; distance C_α_ - C_α_ of 473 and 474 = 3.8 Å) and coexist in six sequences. Finally, positions 270 and 271 are positioned next to each other with concurrency in five sequences.

## Discussion

In this study, we used the sequence variation between different mumps virus strains to assess whether this results in alterations in the structure of the mumps HN and F proteins that could contribute to the occurrence of the recent mumps outbreaks in vaccinated populations. Using homology models of the structure of the F and HN proteins, we compared the amino acid sequences of the genotype A vaccine strains with genotype G strains isolated during recent outbreaks in The Netherlands. We included historic genotype strains as well as more ancient lineages of mumps viruses that had circulated in the Netherlands during the pre-vaccination era to check for genotype-specific variations only linked to either vaccine genotype A or genotype G mumps virus strains, which might explain new mumps outbreaks.

For the F protein, we found some variation, both for the genotype A and the genotype G strains. However, the positions that show variation were mostly located in regions not associated with known functional domains, or in regions with as yet unknown functional properties or were found only in a limited number of sequences. The variation found at position 97 in genotype G may be of interest, as this variant is located near the cleavage site of the F protein. The protease furin cleaves the F protein at this particular site, to expose the fusion peptide after some conformational changes during the fusion process. The exposure of the fusion peptide is essential for fusion of the virus with the host cell membrane^29^. The change at position 97 (S → L) might enhance the fusion process, by introducing more hydrophobicity in the environment of the fusion peptide.

In the HN protein, variation was mostly found in relation to known B-cell epitopes or N-glycosylation sites. The eight variable positions that are specific for one of the two genotypes and which are located in five known B-cell epitopes may have an effect on the ability of the antibodies, elicited by vaccination with the Jeryl Lynn vaccine, to recognize epitopes of genotype G viruses. This was also suggested previously by May *et al*., who showed that certain predicted epitope regions are divergent between the JL vaccine strain and wild type Dutch genotype G strains^16^. However, they only predicted possible B-cell epitopes, whereas we based the regions on literature, which points to empirically proven epitopes. This results in the discrepancy that our study showed a possible relevant variation at positions 354 and 356. May *et al*. seemingly did not find these positions as variable. Additionally, according to our sources, these variations are located in a B-cell epitope, whereas May *et al*. did not predict this region as an antigenic site. Our study adds to this because we also examined the structures of both main surface proteins, as well as surface accessibility of the consensus and variable residues, in contrast to the study by May *et al*. where they only included secondary structure predictions. The structural information has an added value because the protein structures might reveal clustering of variable amino acids that are seemingly located distinctly from each other in the sequence, as seen for the HN protein, as well as to reveal clustering of functional regions of the protein. Clustering of variable positions, might have a cumulative impact on the protein structure and therefore antibody recognition. Additionally, we also analyzed more genotype G strains and compared variable positions to other mumps virus genotypes. This helped us to obtain a more general overview of the extent of variations throughout the genotype A or genotype G sequences as well as to define genotype-specific variations. The eight positions that we showed here to be variable and located in five known B-cell epitope regions should be further investigated. Although some positions displayed a conservative (i.e. similar physicochemical properties between amino acids) variation or were not surface-exposed, they may still have an effect on the tertiary structure. Positions 354 (Q → P), 356 (D → E) and 442 (S → Y) may be of special interest as these variations are specific for the genotype A sequences, both compared to genotype G as well as the other wild type sequences. Additionally, all three variants are surface exposed according to our analysis and the variation in positions 354 and 442 is non-conservative (Fig. 3B). Also position 113 can be of interest because this variation is specific to genotype G sequences and is located in a known B-cell epitope. Positions 113, 354, 356 and 442 deserve further investigation because either all genotype A or genotype G sequences showed this variation unlike positions 129, 205 and 330. These variable positions were found to be located in a B-cell epitope and/or an N-glycosylation site, which might change the recognition site for vaccine-induced antibodies, by loss of the N-glycosylation site (position 129). However, as this variation only appears in one sequence, it is most likely a random variant, and hence not genotype-specific. It is important to note that positions 113, 354, 356 and 442 are particularly interesting as it was shown for other viruses such as influenza, that changes in as few as one amino acid located in a B-cell epitope can lead to reduced antibody recognition^34^. This observation can explain why a single amino acid change in a B-cell epitope can lead to the reduction or loss of recognition of this epitope by vaccine-induced antibodies. Additionally, this hypothesis is supported by the finding that antibodies induced by the mumps vaccine (JL, genotype A) are less potent in neutralizing wild type virus than the vaccine virus (Rubin *et al*. 2008; Dayan & Rubin 2008; Gouma *et al*. 2016)^12,23,24^. In our study, we found variation in eight positions located in five different B-cell epitopes. The four positions mentioned above (113, 354, 356 and 442) might be relevant and should be subject of further investigation to examine the influence of these variations on antibody recognition and viral neutralization.

We also found a small number of differences in or near other important functional regions of the HN protein. These variants are mostly conservative, which suggests that these positions do not tolerate much change most likely due to their primary function of virus-host cell fusion. For example, positions 203 and 205 in the HN protein changed from K to N and H to R in a small number of genotype G and genotype A sequences, respectively. These conservative variants are located near a neuraminidase region, which is an important functional region of the HN protein. In addition, we have not found any variable sites in regions important for Ca^2+^-binding, which plays a role in structural stability and these sites are therefore most likely conserved^35^.

**Figure 4:**
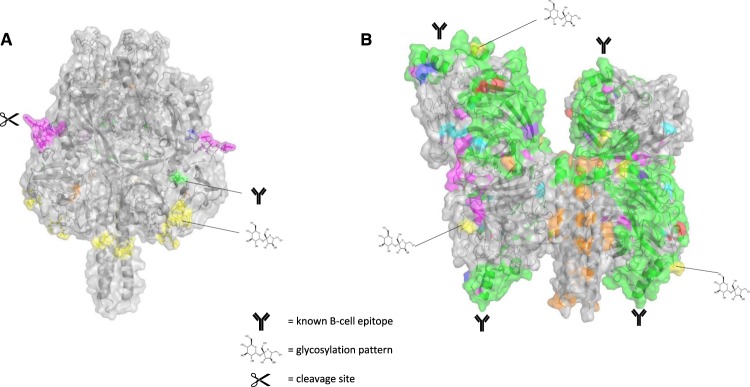
*In silico* protein model of the F and HN protein with the specific functional regions mapped on the structures in different colors. (**A**) F protein with specific regions which are colored as described in the legend to Fig. 3. (**B**) HN protein with specific regions which are colored as described in the legend to Fig. 3.

Some of the variable positions found in this study were indeed empirically verified before. The variation found at position 354 in the HN protein, which is located in a known B-cell epitope, has also been described by other research groups^26,36,37^. Most of the variants found in the HN protein encompass the variable regions described in the study by Vaidya *et al*., in which they compared mumps virus genome sequences from two genotype G and six genotype C strains, with several vaccine strains, including the Jeryl Lynn strain^38^. Additionally, we show that these regions, except for regions 240–245 and 405–410, are surface exposed (Fig. 4). The antibody recognition of the protein surface might alter when certain residues become surface exposed or buried when variation occurs, which might lead to loss of protection. We found variants at positions 279 and 287 (change of T to I and I to V, respectively) of the HN protein, which has also been reported previously^25^. This variation can generate a mismatch in recognition of CD4^+^ T-cell epitopes, between genotype A and genotype G strains. This may lead to the loss of an important T-cell response, which supports the immunological recognition and recall response. Positions 279 and 287 can both, individually and additively, lead to a difference in T-cell responses against the Jeryl Lynn vaccine strains and other wild type strains, as recognition of the T-cell epitope by the HLA molecules may be altered^25^. The study by Dilcher *et al*. showed that the above-mentioned interesting positions 279, 287, 336, 356 and 442 indeed are related to known B-cell epitopes^39^. With their structural comparison they also concluded that differences between genotype A and genotype G mumps viruses might have an influence on antibody recognition in these B-cell epitope regions.

In summary, despite the fact that the F and HN proteins of mumps viruses of genotype A and G are very similar, we have found a series of amino acid variations between these proteins of genotype A vaccine strains and genotype G wild type strains isolated from infected individuals. Most of the observed differences are located in regions of functional importance or in known B-cell epitopes. Therefore, it would also seem relevant to base mumps genotyping on the HN or F gene, instead of SH, as the differences in the HN or F gene are more relevant for the evolution of the virus, due to immunological pressure. It was shown previously that HN and F gene based genotyping is better suited for recording virus transmission^27^. The importance of appropriate genotyping was also recently shown by Dilcher *et al*., who reported a modified next generation sequencing protocol for better mumps diagnosis, variation analysis and outbreak control^39^. Eight variants specific for genotype A or G strains were located in five different known B-cell epitopes of the HN protein. These changes may lead to a reduced recognition of genotype G strains by vaccine-induced immune responses. This reduced recognition of B cell epitopes and the ensuing reduction of protective capacity of the vaccine-induced antibodies could thus contribute to the recent outbreaks of genotype G mumps in persons vaccinated with the genotype A JL vaccine. Functional assays are needed to corroborate our results and provide a conclusive answer to the question why mumps is resurgent in vaccinated populations.

## Methods

### Sequences

For this analysis, a total of 184 and 231 sequences were used for the F and HN protein, respectively. Of the 184 sequences of the F protein, 28 sequences were retrieved from GenBank, *i*.*e*. 9 sequences of genotype A, 8 sequences of genotype G and 11 sequences of other genotypes (B, C, F, H and K; Supplementary Table 1). The remaining 156 sequences (46 pre-vaccine samples, 110 genotype G samples) were derived from patient samples collected at the RIVM. The 46 pre-vaccine samples were obtained between 1957 and 1982. Selection of the mumps virus strains was based on availability of the isolates. According to the available information for these isolates, mumps virus had been cultured from oral swabs, nasal swabs and cerebral spinal fluid samples. All but one of the viruses were isolated from Dutch mumps patients. The non-Dutch isolate was from a patient from Albany, USA. These 46 sequences contained genotypes A, D, L and an unclassified genotype. The mumps virus genotype G sequences from 110 clinical samples obtained between 2004 and 2015 were also sequenced.

Similarly, 76 of the 232 sequences of the HN protein were retrieved from GenBank, *i*.*e*. 11 sequences of genotype A, 22 sequences of genotype G and 43 sequences of other genotypes (B, C, D, F, H, I, J, K and L; Supplementary Table 1). For each genotype at least one reference strain for that genotype was included^17^. The remaining 156 sequences were also derived from patient samples, as was done for the F protein, *i*.*e*. 46 sequences were pre-vaccine samples (genotype A, D, L and unclassified) and the other 110 sequences were genotype G sequences. For phylogenetic analysis, the SH gene sequences of 78 mumps virus strains were retrieved from GenBank (Supplementary Table 1). The other 156 samples (46 pre-vaccine strains, 110 genotype G strains) were sequenced. The sequences of the 156 samples for the F, HN and SH proteins, have been described previously, except for one (Gouma *et al*. 2016). The GenBank accession numbers are: KJ125045-KJ125051, KJ125053-KJ125059, KJ125061-KJ125067, KU756625-KU756710, KU756712-KU756812, KU756814-KU756914, KU756916-KU756930, and KX136898-KX137038. As the Jeryl Lynn strain is a mixture of different strains, all sequences of the different Jeryl Lynn strains were included. In accordance with the Dutch law, no informed consent was required for this study.

### Sequencing and phylogenetic analysis

RNA extraction, cDNA synthesis and sequencing of the F gene, SH gene and HN gene for all genotype G mumps virus strains were performed as described previously (Gouma *et al*. 2016)^12^. For sequencing of the strains from the pre-vaccination era, the same procedures were followed for the sequencing of the F and SH gene for the genotype G strains, as previously described (Gouma *et al*. 2016)^12^. For sequencing of the HN gene, cDNA was synthesized with the use of primer FW-HN1 (nt 6535–6555) and thereafter a PCR was performed with primers FW-HN1 and RV-HN1 (nt 8442–8460). The six primers used for sequencing were FW-HN2 (nt 6539–6557), FW-HN7172 (nt 7172–7191), RV-HN7233 (nt 7212–7233), FW-HN7795 (nt 7795–7814), RV-HN7842 (nt 7823–7842) and RV-HN2 (nt 8435–8454) as described previously^40^. BioNumerics software version 7.5 (Applied Maths) was used to analyze nucleotide sequences and to recreate a maximum parsimony tree with bootstrap resampling (1000 replicates) based on the SH gene sequences.

### Generation of the homology models

Homology models were generated for both the F and HN proteins, as there were no experimentally determined structures of the mumps F and HN proteins available, at the time of analysis. The consensus sequence was calculated for both the F and HN proteins based on 123 and 196 sequences, respectively, with the cons module of Emboss^41^. This consensus sequence was then used in a BLAST search^42^ against the Protein Data Bank (PDB)^43^ to identify available structures of homologs for both proteins. Overall, PDB-entries 4GIP^44^ and 3MAW^45^ were used as templates for the F protein in the pre- and post-fusion conformation, respectively, as the F protein undergoes a conformational change during the fusion process^29^. PDB-entry 4GIP^44^ contains the structure of the F protein from parainfluenza virus 5 and has an identity and similarity score with the mumps F protein consensus sequence of 49% and 69%, respectively; whereas PDB-entry 3MAW^45^ contains the structure of the F protein from Newcastle Disease virus and has an identity and similarity score of 35% and 54%, respectively. PDB-entry 1Z4V^46^ was selected as template for the HN protein. This structure contains the HN protein from parainfluenza virus 5 and has an identity and similarity score with the mumps HN protein consensus sequence of 46% and 66%, respectively.

After selection of the templates, FoldX^47^ was used to replace the amino acids of the template with the mumps F and HN protein consensus sequences. Next, an energy minimization was carried out in YASARA with the YASARA force field^48^, to find the most stable local minimum of the protein conformation. Finally, the models were evaluated by MolProbity^49^, to avoid steric hindrance and side chain clashes.

For the F protein, positions 1–19, 101–102 and 478–538 are absent in the structure. For the HN protein, positions 1–79 and 119–127 are absent in the structure. These missing structures are signal peptides, located inside the virion, or are the transmembrane part of the protein.

### Multiple sequence alignment and mapping of the mutations

Multiple sequence alignments for the F and HN protein sequences were performed with the aid of Muscle^50,51^. The sequence variation was subsequently mapped onto the protein structures with the aid of Scop3D, a tool to visualize variation across multiple sequences on the protein structure^28^. The entropy, which provides information on how random or specific the observed variation is, was also calculated with Scop3D for both proteins. F protein numbering is based on GenBank accession number JN012242 and HN protein numbering is based on accession number ABY81903.

### Determination and visualization of variable positions and specific functional regions

Specific functional regions were defined based on literature (Table 1). An N-glycosylation site is defined as the glycosylation recognition pattern N-X-S/T with X being any amino acid except P. All regions were subsequently mapped onto the sequences and models of the protein structures for analysis (Fig. 4), analyzed for diversity, and visualized on the structures of the homology models with the aid of PyMol (PyMOL Version 11r1, Schrödinger LLC). Numbering of the positions is based on GenBank accession number JN012242 and GenBank accession number ABY81903, for the F and the HN protein respectively.

### Calculation of the solvent-accessible surface area

The absolute solvent-accessible surface area (aSAS), the area of a residue that is accessible by a water molecule (radius of 1.4 Å), was calculated with the aid of DSSP^52,53^. The relative solvent-accessible surface (rSAS) area was then obtained by calculation of the ratio of the aSAS of the amino acid as present in the protein to the aSAS of the residue in the tripeptide G-X-G^54,55^. A residue is said to be surface-accessible (solvent exposed) if the relative SAS is >25%, otherwise a residue is said to be buried^56,57^. A surface exposed residue can be targeted by other molecules, such as antibodies. The RMSD of the homology model and the experimentally defined structure of the HN protein was calculated by using the Align command in PyMol (The PyMOL Molecular Graphics System, Version 1.1r1, Schrödinger, LLC).

## Electronic supplementary material

Supplementary information
