# Publisher Correction: Differences in antigenic sites and other functional regions between genotype A and G mumps virus surface proteins

**DOI:** 10.1038/s41598-020-62301-7

**Published:** 2020-03-20

**Authors:** Sigrid Gouma, Tessa Vermeire, Steven Van Gucht, Lennart Martens, Veronik Hutse, Jeroen Cremer, Paul A. Rota, Geert Leroux-Roels, Marion Koopmans, Rob van Binnendijk, Elien Vandermarliere

**Affiliations:** 1 Centre for Infectious Disease Control, RIVM, Bilthoven, The Netherlands; 2 Department of Viroscience, Erasmus MC, Rotterdam, The Netherlands; 3 Microbiology Department, Perelman School of Medicine, University of Pennsylvania, Philadelphia, USA; 4 National Reference Centre for Measles, Mumps and Rubella, Sciensano, Brussels Belgium; 5 Department of Biochemistry, Ghent University, Ghent, Belgium; 6 VIB-UGent Center for Medical Biotechnology, VIB, Ghent, Belgium; 7 National Center for Immunization and Respiratory Diseases, Centers for Disease Control and Prevention (CDC), Atlanta, USA; 8 Center for Vaccinology, Ghent University, Ghent, Belgium

Correction to: *Scientific Reports* 10.1038/s41598-018-31630-z, published online 06 September 2018

In the original version of this Article, the author Rob van Binnendijk was incorrectly indexed. This error has now been corrected.

