# Supplementary material for: Differences in antigenic sites and other functional regions between genotype A and G mumps virus surface proteins
Source: Sci Rep. 2018 Sep 6;8:13337. doi: 10.1038/s41598-018-31630-z (PMC6127219; doi:10.1038/s41598-018-31630-z)
Supplement: Supplementary file 1 — Supplementary information [file 41598_2018_31630_MOESM1_ESM.docx]

Differences in antigenic sites and other functional regions between genotype A and G mumps virus surface proteins

Sigrid Gouma^1, 2, 3#^, Tessa Vermeire^4, 5, 6#^, Steven Van Gucht^4^*, Lennart Martens^5, 6^, Veronik Hutse^4^, Jeroen Cremer^1^, Paul A. Rota^7§^, Geert Leroux-Roels^8^, Marion Koopmans^1,2^, Rob van Binnendijk^1^, Elien Vandermarliere^5,6^

^1^Centre for Infectious Disease Control, RIVM, Bilthoven, the Netherlands; ^2^Department of Viroscience, Erasmus MC, Rotterdam, the Netherlands; ^3^Microbiology Department, Perelman School of Medicine, University of Pennsylvania, Philadelphia, USA, ^4^National Reference Centre for Measles, Mumps and Rubella, Scientific Institute of Public Health (Sciensano), Brussels, Belgium; ^5^Department of Biochemistry, Ghent University, Ghent, Belgium; ^6^VIB-UGent Center for Medical Biotechnology, VIB, Ghent, Belgium; ^7^National Center for Immunization and Respiratory Diseases, Centers for Disease Control and Prevention (CDC), Atlanta, USA; ^8^Center for Vaccinology, Ghent University, Ghent, Belgium

# these authors contributed equally to this work

*Corresponding author; steven.vangucht@sciensano.be

^§^The findings and conclusions in this report are those of the authors and do not necessarily represent the official position of the Centers for Disease Control and Prevention.

***Supplementary table 1.*** *Overview of the GenBank sequences used for phylogenetic analysis and for analysis of the F and HN proteins.*

|  | **GenBank accession number** | | |
| --- | --- | --- | --- |
| **Genotype** | **SH gene** | **F gene** | **HN gene** |
| A | AF201473, AF338106*, AF345290, FJ211586, FN431985, GU980052*, HQ416906, HQ416907 | AF201473, AF338106*, AF345290, AJ010821, AJ133693, FJ211586, FN431985, HQ416906, HQ416907 | AF201473, AF338106*, AF345290, AY584603, AY584604, FJ211586, FN431985, HQ416906, HQ416907, X93178, X93179, |
| B | AB000388*, AB823535, AB827968, JQ945269* | AB823535, AB827968 | AB823535, AB827968, JQ946041* |
| C | AY669145, EU370206*, JQ034465, JQ034466, JQ945268* | AY669145 | AY669145, JQ034465, JQ034466, JQ999999* |
| D | JQ034452*, JQ945275* |  | JQ034464*, JQ946039* |
| F | DQ649478, EU780221*, EU884413, FJ556896, HQ693823, HQ693825, JQ034459, JQ034460, JQ034461, JQ945272* | EU884413, FJ556896 | DQ649478, EU884413, FJ556896, HQ693823, HQ693824, HQ693825, HQ693826, JQ034459, JQ034460, JQ034461, JQ034462, JQ034463*, JQ946034* |
| G | AF280799*, EU370207, EU597478*, JN012242, JN635498, JX287385, JX287387, JX287389, JX287390, JX287391, JX390987, JX390988, JX390989, JX390990, JX390991, JX390992, JX390993, JX390994, JX390995, JX878447, KF481689 | JN012242, JN635498, JX287385, JX287387, JX287389, JX287390, JX287391, KF481689 | EU370207, JN012242, JN635498, JQ946046*, JX287385, JX287387, JX287389, JX287390, JX287391, JX390987, JX390988, JX390989, JX390990, JX390991, JX390992, JX390993, JX390994, JX390995, JX878447, KC852187, KC852188, KF481689 |
| H | AB600843*, AF467767, AY681495, JN687469, JQ388690, JQ388691, JQ945273*, JX287388 | AF467767, AY681495, JQ388690, JQ388691, JX287388 | AF467767, AY681495, JN687469, JQ388690, JQ388691, JQ946035*, JX287388 |
| I | JQ945274*, AY309060* |  | JQ946037* |
| J | JQ945271*, AB105475* |  | JQ946033*, JQ946044* |
| K | EU082458, JQ945276*, JQ945270*, JX287386, KC921200, KC921201, KC921202, KC921203, KF212191 | JX287386 | JQ946040*, JQ946045*, JX287386, KC921200, KC921201, KX921202, KX921203, KF212191, JF268685 |
| L | AB105483*, AB105480* |  | JQ946036*, JQ946043* |
| N | AY508995*, AY685920* |  |  |
| Unclassified | AF142774*, AB003415*, AY380077* |  |  |

** WHO reference strain*
